# The Role of Genetics in Sex Diversity

**DOI:** 10.1515/medgen-2023-2035

**Published:** 2023-08-16

**Authors:** Olaf Hiort, Ulrike Krämer, Lisa Malich, Christoph Rehmann-Sutter, Malte Spielmann

**Affiliations:** Klinik für Kinder- und Jugendmedizin Ratzeburger Allee 160 23562 Lübeck Deutschland; Klinik für Neurologie Lübeck Deutschland; Universität zu Lübeck Lübeck Deutschland; Universität zu Lübeck Königstraße 20 23552 Lübeck Deutschland; University Medical Center Schleswig-Holstein, University of Lübeck & Kiel University, Institute of Human Genetics Ratzeburger Allee 160 23562 Lübeck Deutschland

Sex is a major and complex feature of individual development and concerns all levels of the human appearance – from cells to tissues to hormones to organs to behaviour. It marks embodiment and is the reference for gender. And it may also be the most controversial feature of “the” human body. In recent years, scientific knowledge on the diversity of expression patterns of sex beyond typical femaleness and maleness in human biology has vastly increased. However, in clinical medicine and in cultural attitudes, sex is still very often reduced to the usual binary categories of male and female. Moreover, the categorization into male and female is taken as a given and without a frame of reference. It is seen as a natural fact without further questions about its ontological basis, about the underlying cultural assumptions, and about necessary or possible distinctions within and between the ‘two’. Therefore, the (scientific) understanding of sex is hampered by a traditional doctrine which does not relate to the biological diversity of sex in bodily expression, shaped by an intricate genetic and hormonal regulation. A growing attention to sex diversity and its societal recognition has only partly been connected with biological knowledge, biomedical research and human genetic practices. 

In this issue of *Medizinische Genetik*, we compiled four manuscripts describing the current scientific understanding of sex and sex diversity. The article by Hornig et al. describes the “past and future of sex genes”, taking a hormonal perspective, which can be traced back to the emergence of biochemical endocrinology in the last century. At the same time, chromosomal structures became known, and towards the end of the century molecular genetics defined “master switches” of sex development with a dominant male and a weaker female. The authors thus trace the history of how this culturally biased perception, based on a supposed binarity of sex with apparent “male dominance”, developed and became established in much of biomedicine. In contrast, Holterhus et al. explain the current view of the 21^st^ century, when the terminology of “master switches” is losing out against the perception of a complex regulatory network that controls sex expression both through gene expression patterns as well as hormonal profiles. Rehmann-Sutter and co-authors therefore ask: “Is sex still binary?”. Their paper links the scientific gain of knowledge about the multi-factorial and multi-dimensional aspects of sex development with changes in social and legal perspectives on sex. In 2006, a much-noticed medical consensus conference had defined conditions of humans with variances between chromosomal, gonadal, and phenotypic sex as “DSD”, initially seen as Disorders of Sex Development and nowadays described as *Differences* of Sex Development. This was meant as a scientifically better defined alternative to previously used terminology such as “intersex” or “hermaphroditism”. Today, however, some people with DSD use these terms in an affirmative way to describe themselves. In Germany, activists have initiated a ground-breaking change in personal status law with the acknowledgment of a third option of sex in addition to male and female, named “diverse”. This change has profound impact on the socio-cultural perception of sex and provides a first link between the recognition of biological sex variability and appreciation of socio-cultural sex perception. The changes in medical care of people with DSD is the topic of the fourth article provided by Kulle and colleagues. They stress that not only the attitude of health care professionals has to change when managing patients with DSD, but also the assessment of laboratory and technical values altogether has to be adapted. 

Overall, we want to encourage the readers of this issue to acknowledge the complexity of perspectives on sex and gender in the modern world, and to emphasize their implications for the medical research environment. Recognising and investigating the diversity of sexes beyond unquestioned binarity may have profound effects on understandings of sex related phenomena and also on scientific research practices as well as on wider society. We believe that in the future, the emerging understandings need to be systematically studied from diverse disciplinary angles and stakeholder perspectives, and through a multitude of interlinked methodological approaches. Only then we will gain insight into the relevance of sex diversity for various bio-medical issues. Furthermore, there will a chance to connect the biomedical sciences to the equally rapidly changing socio-cultural perception of sex and gender.

